# On the Use of Astringent and Stimulating Injections in Profuse Suppuration

**Published:** 1847-12

**Authors:** Daniel Brainard

**Affiliations:** Professor of Surgery in Rush Medical College


ARTICLE VIII.

*On the use of astringent and stimulating injections in profuse suppuration.* By DANIEL BRAINARD, M. D., Professor of Surgery in Rush Medical College.

Every practical surgeon must have met with cases of suppuration following upon erysipelatous or morbid inflammation, in which the tissues seem to melt away, or be dissolved in pus, and those who have the care of hospitals, or have practiced where the epidemic erysipelas was prevalent, can testify to the frequency with which this abundant suppuration carries off patients; those, especially, who have lost much blood from wounds or operations, or who are addicted to the use of alcoholic drinks, or debilitated from previous disease, when attacked with diffuse suppuration, are speedily exhausted by the discharge, if not poisoned by the absorption of pus into the system.

Under these circumstances, the ordinary means recommended are often, if not generally ineffectual; for wine, bark, brandy, acids, and nourishing soups, although they support the strength and prolong life, are, for the most part, but partial palliatives, for they can neither restore the waste of blood from copious suppuration, nor prevent purulent absorption or its effects, nor indeed be retained in considerable quantities in the stomach. Hence it becomes necessary to resort to other means, and that which we would recommend to the profession is the direct application of stimulant and astringent solutions to the purulent surface.

Pus is a fluid containing corpuscles analagous to those of the blood, rich in the nutritive portions of that fluid, and when lost in large quantities, is attended with the same results as hemorrhage or profuse serous diarrhœa. If the term serous hemorrhage be applicable to this latter disease, that of purulent hemorrhage is much more suitable for these cases which we are at present considering, and since, in an oozing of blood from a mucus or cut surface, or in a serous discharge, we resort at once and with promptness to local astringents, or the internal use of acetate of lead and opium, so in case of exhausting suppuration we should use powerfully astringent and stimulating applications directly to the pus forming surface—astringent for the purpose of checking its formation, and stimulant for the purpose of exciting adhesive inflammation, preventing the diffusion of pus among the tissues, and guarding against its resorption by the vessels. In this latter we have an important danger to guard against, which we do not fear either in cases of hemorrhage or effusions of serum.

After having, during several years, in which erysipelas and extensive suppuration were prevalent, seen patients sink more or less rapidly in spite of other means, we determined to put the above views into practice in a case of an extremely hopeless character which came under our care.

Oliver Benson, a healthy sailor, of 31 years of age, received, on the 9th of Nov., 1847, a severe compound fracture of the right leg, three inches above the ankle, attended with much laceration of the skin, contusion of the muscles, comminution of the bones, and rupture of the anterior tibial artery and nerve. Amputation was judged necessary, and performed immediately, about four inches below the knee, in presence of the medical class; but before this was resorted to he was already greatly exhausted from loss of blood. During the following night he was restless, and moved the stump so violently as to cause some hemorrhage. For the two succeeding days he was restless, suffering pain in the stump and various parts of the body. Pulse quick; tongue coated. On the third day the stump seemed to be nearly healed by the first intention, and was not inflamed, but on the morning of the fourth an erysipelas was perceptible around the wound. A cathartic of calomel was administered, and evaporating lotions applied to the inflamed part. The disease, however, was not checked, but extended up, on the outside, above the knee, and on the third day from its commencement, fluctuation was perceived about three inches above the knee. The abscess having opened a copious discharge of pus took place. The next day, the abscess had spread upward above the middle, and upon the under side of the thigh, and the suppuration was still more abundant. Two other openings were made, and compresses and a roller applied. This did not arrest it; the erysipelas extended to the hip and side of the body, and the suppuration to the pelvis, and sloughs were drawn out from the openings. While the disease was making this progress, the symptoms became aggravated, with prostration, diarrhœa, and occasional vomiting, for which, opium, and acetate of lead, wine, capsicum, and animal broths were given to the extent of tolerance, but without effect.

At this period we made a saturated solution of alum in water, to which was added four grains of sul. copper to the ounce, and after evacuating the pus by free orifices, the whole cavity of the absess was filled with this solution, and the orifices being closed, it was retained until the smarting and pain were severe, and then allowed to escape, and pure water injected to wash it out. The roller and compresses were then applied. The immediate result was the reduction of the discharge from twenty ounces, to three or four, in twenty-four hours, and this was of healthy consistence.

The suppuration extended no further, and the progress of the disease was arrested. The injections were repeated daily for a week. The stump which had commenced to slough, assumed a healthy appearance, and the patient became speedily convalescent.

The principle upon which the practice is based, is the same as that upon which blisters are used in similar cases, viz: exciting a healthy inflammation; but the injections have the additional advantage of arresting the discharge, and producing in the walls of the abscess an adhesive process, by which its progress is arrested.

The efficacy of such injections into purulent cavities connected with scrofulous joints, has long been familiar to us, and some illustrations of its effects in such cases, were given in a former number of this journal, under the head of amputation in such cases. Our further reflections and experience have led us to recommend its use in all those cases in which the system is exhausted by purulent formations from the blood, beyond the power of the nutritive process to supply. There are great numbers of these cases in which death is not caused by disease of vital organs, but by the drain upon the system. Such are the diffuse suppurations from erysipelas, from abscesses near carious joints, from disease of the spine, as in lumbar and psoas abscesses, and in extensive deposits from purulent absorptions.

These being all either very dangerous, or entirely hopeless cases, under the best treatment hitherto pursued, the discovery of additional means of resisting them is greatly to be desired, and it is with the hope that the practice here recommended may be of this kind, that these observations are submitted to the profession.

